# Occurrence and Antimicrobial Susceptibility Profile of *Salmonella* spp. in Raw and Ready-To-Eat Foods and *Campylobacter* spp. in Retail Raw Chicken Meat in Transylvania, Romania

**DOI:** 10.1089/fpd.2019.2738

**Published:** 2020-08-06

**Authors:** Emil Tîrziu, Gabriel Bărbălan, Adriana Morar, Viorel Herman, Romeo T. Cristina, Kálmán Imre

**Affiliations:** ^1^ Department of Animal Production and Veterinary Public Health, Faculty of Veterinary Medicine, Banat's University of Agricultural Sciences and Veterinary Medicine “King Michael I of Romania”, Timişoara, Romania.; ^2^ Department of Infectious Diseases and Preventive Medicine, Faculty of Veterinary Medicine, Banat's University of Agricultural Sciences and Veterinary Medicine “King Michael I of Romania”, Timişoara, Romania.; ^3^ Department of Pharmacology and Pharmacy, Faculty of Veterinary Medicine, Banat's University of Agricultural Sciences and Veterinary Medicine “King Michael I of Romania”, Timişoara, Romania.

**Keywords:** *Salmonella*, *Campylobacter*, food, antimicrobial resistance, Romania

## Abstract

The survey was undertaken to investigate the presence and antimicrobial susceptibility profile of *Salmonella* spp. in raw and ready-to-eat (RTE) foods, and *Campylobacter* spp. in the retail raw chicken meat collected in two counties of Transylvania, Romania. A total of 13.1% (51/388) of the examined food samples were found to be *Salmonella* positive, with a distribution of 14.7% (48/326) in the raw food (i.e., pork, chicken carcass, and shell egg) and 4.8% (3/62) in the RTE samples (i.e., sausages, but not ham and salami), respectively. These differences were statistically significant (*p* = 0.034). The isolates were serotyped as *Salmonella* Infantis (*n* = 19), *Salmonella* Typhimurium (*n* = 11) *Salmonella* Rissen (*n* = 8), *Salmonella* Derby (*n* = 3), *Salmonella* Enteritidis (*n* = 3), *Salmonella* Bredeney (*n* = 2), *Salmonella* Brandenburg (*n* = 1), *Salmonella* Gloucester (*n* = 1), *Salmonella* Goldcoast (*n* = 1), *Salmonella* Kottbus (*n* = 1), and *Salmonella* Ruzizi (*n* = 1). *Campylobacter* strains were present in 29.4% (10/34) of the investigated chicken samples, and the identified species were *Campylobacter coli* (70%) and *C. jejuni* (30%). From the 14 tested antimicrobials, the *Salmonella* isolates were resistant against azithromycin (88.2%), tetracycline (54.9%), sulfamethoxazole (54.9%), ciprofloxacin (45.1%), nalidixic acid (43.1%), ampicillin (35.3%), chloramphenicol (33.3%), tigecycline (25.5%), cefotaxime (13.7%), colistin (13.7%), trimethoprim (7.8%), and gentamicin (2%), resulting in the expression of 21 multidrug-resistant (MDR) profiles. Of 10 *Campylobacter* isolates, 80% were resistant to ciprofloxacin and nalidixic acid, 40% to tetracycline, and 10% to streptomycin and erythromycin, respectively. Our findings indicate that Romanian isolates of *Salmonella* spp. and *Campylobacter* spp., contaminating animal-origin foods, can exhibit MDR patterns, representing a public health risk.

## Introduction

*Salmonella* spp. and *Campylobacter* spp. are recognized as two of the most important foodborne pathogens that can cause severe infections in humans and economic losses worldwide. Their presence is monitored in different steps of the food chain, and especially in finished raw and ready-to-eat (RTE) products, as a safety criterion for the consumer, representing a very important tool for implementing efficient food safety systems (Antunes *et al.*, 2016; Khan *et al.*, 2018).

In recent years, the large-scale overuse of common antimicrobials in human and veterinary medicine with different purposes (e.g., therapeutics, prophylactics, and growth promoters) have accelerated the emergence and spread of antibiotic-resistant foodborne bacteria. Nowadays, the antimicrobial resistance (AMR) phenomenon is considered one of the most worrisome public health concerns, with negative impact on the effectiveness of public health interventions (Zhang *et al.*, 2018; European Food Safety Authority, 2019).

The European Union (EU) member states make great efforts to establish harmonized interinstitutional strategies under a One Health approach to combat AMR. The collection of comparable, up-to-date, and reliable data is a prerequisite for the implementation of effective risk management measures by assessors. In this regard, baseline information about the occurrence of AMR zoonotic bacteria from the human–animal–food interface in Europe is published yearly by the European Food Safety Authority (EFSA) and European Center for Disease Prevention and Control, within a summary report (European Food Safety Authority, 2019), with the contribution of each member state. In the most recent EFSA report, data provided by Romania revealed a total of 1154 (with 5.9 notification rates per 100,000 populations) and 467 (with 2.4 notification rates per 100,000 populations) human confirmed cases of salmonellosis and campylobacteriosis, respectively.

Data provided by Romania regarding the overall AMR profile of food origin pathogens refers especially to strains isolated from raw pork, beef, and poultry meat, without offering any supplementary information about their origin, according to the types of finished products, and in association with detailed antibiotic susceptibility profile data of the implicated species and/or serotypes. In addition, in the context of the importance of the global fight against AMR, the number of scientific reports on *Salmonella* and *Campylobacter* in our country are quite limited (Mihaiu *et al.*, 2014; Dan *et al.*, 2015; Morar *et al.*, 2015; Tîrziu *et al.*, 2015), and additional studies are still required.

This study aimed to provide data on the occurrence and antimicrobial susceptibility profile of two major foodborne pathogens (*Salmonella* spp., *Campylobacter* spp.) in different food products, from two Transylvanian counties of Romania.

## Materials and Methods

The study was undertaken between 2016 and 2018 in two counties of the historical Transylvania region, located in central Romania. A total of 292 raw food samples of pork (*n* = 146), chicken carcasses (*n* = 98), and shell eggs (*n* = 48; deriving from whole chicken eggs) provided from private production units were screened for the presence of *Salmonella* spp. within the self-control process of each batch, in conformity with Regulation (EC) 1441/2007 (EC 1441/2007). Similarly, 62 RTE products, including sausages (*n* = 37), ham (*n* = 9), and salami (*n* = 16), and fresh chicken meat samples (*n* = 34), randomly collected from retail markets by veterinary inspectors within the official organized controls, were analyzed, to detect *Salmonella* spp. In addition, the retail chicken samples were monitored for the presence of *Campylobacter* spp.

In nine sampling days of the first month of each trimester of the calendar year, a total of 17 randomly selected different retail markets of surveyed area were visited. On the sampling day of each of these from two to four food matrices providing from different batches was sampled. The samples were collected using sterile gloves in sterile bags and labeled. On the same day, the specimens were transported in an isothermal box to the Food Microbiology Laboratory of the Sanitary Veterinary Directorate of each county.

*Salmonella* spp. and *Campylobacter* spp. isolates were detected using the national standardized methods SR EN ISO 6579/2003 AC/2006 (Romanian Standards Association, 2003) and SR EN ISO 10272/2006 (Romanian Standards Association, 2006), respectively. In brief, for the isolation of *Salmonella* spp. from the 25 g pre-enriched food matrix, the xylose lysine deoxycholate (Biokar Diagnostics) and Rambach (Biokar Diagnostics) mediums were used and the inoculated plates were incubated afterward at 37°C for 24 h. The isolation of *Campylobacter* spp. from the enriched samples was carried out on Columbia agar supplemented with sheep blood (Oxoid) at 42°C for 48 h under microaerobic conditions. For each positive sample, a single randomly selected bacterial strain was submitted to the Institute for Hygiene and Public Health (București, Romania) for antimicrobial susceptibility testing and serotyping in case of *Salmonella* isolates. Serotyping was carried out in accordance with the Kauffmann–White scheme by microtiter slide agglutination using polyvalent O- and H-antisera (Difco; BD, Detroit, MI).

Antimicrobial susceptibility testing was performed using the Sensititre^®^ microbroth dilution system (Trek Diagnostic Systems, Inc., Cleveland, OH), in accordance with the manufacturer's instructions, and following the International Organization for Standardization [ISO 20776-1:2007 (ISO, 2007)] guideline. For *Salmonella* the EUVSEC (Trek Diagnostics Systems, Inc.) plate was used and the quality control used the *Escherichia coli* ATCC 25922 strain. In case of *Campylobacter* the EUCAMP2 (Trek Diagnostics Systems, Inc.) plate, together with *C. jejuni* 33560, as a quality control strain, was used. For both pathogens, the resistance breakpoints were established according to the Clinical and Laboratory Standards Institute (CLSI, 2018, M100-28) and the European Committee on Antimicrobial Susceptibility Testing (EUCAST, 2017) (for colistin and tigecycline only) guidelines. The isolates were categorized as resistant, intermediate, or susceptible. In this survey, only absolutely but not intermediate resistant strains were considered as resistant isolates.

The tested 14 antibiotics for *Salmonella* strains were provided from 12 classes. The antimicrobial substances and their concentrations included in the panel were as follows: β-lactams—ampicillin (AMP; 1–64 μg/mL), aminoglycosides—gentamicin (GEN; 0.5–32 μg/mL), carbapenem—meropenem (MEM; 0.03–16 μg/mL), cephalosporins—cefotaxime (CTX; 0.25–4 μg/mL), ceftazidime (CAF; 0.5–8 μg/mL), fluoroquinolones—ciprofloxacin (CIP; 0.015–8 μg/mL), glycylcycline—tigecycline (TGC; 0.25–8 μg/mL), polymyxins—colistin (CST; 1–16 μg/mL), macrolide—azithromycin (AZM; 2–64 μg/mL), nitrobenzenes—chloramphenicol (CHL; 8–128 μg/mL), sulfonamides—sulfamethoxazole (SMX; 8–1024 μg/mL), trimethoprim (TMP; 0.25–32 μg/mL), quinolones—nalidixic acid (NAL; 4–128 μg/mL), and tetracyclines—tetracycline (TET; 2–64 μg/mL).

In case of *Campylobacter* spp. the selected drugs belonged to five classes and included the following: fluoroquinolones—CIP (0.12–16 μg/mL); macrolide—erythromycin (ERY; 1–128 μg/mL), GEN (0.12–16 μg/mL); quinolones—NAL (1–64 μg/mL), aminoglycosides—streptomycin (STR 0.25–16 μg/mL), and tetracyclines—TET (0.5–64 μg/mL). The isolates were considered multidrug-resistant (MDR) if they exhibited nonsusceptibility to at least one antimicrobial in three or more antimicrobial classes (Magiorakos *et al.*, 2012).

The statistical analysis of the frequency of isolation of pathogens in relation to their sample origin was performed with the Pearson's chi-square (χ^2^) test (Microsoft Excel 2007, Redmond, WA), and a value of *p* ≤ 0.05 was considered significant.

## Results

Altogether, 13.1% (51/388) of the examined food samples were found to be *Salmonella* positive. The overall prevalence of *Salmonella* spp. in raw food samples was 14.7% (48/326), and its frequency of isolation according to the tested products was 22.6% (33/146) in pork, 9.1% (12/132) in chicken (9.2%—9/98 of carcasses and 11.8%—4/34 fresh retail meat), and 6.3% (3/48) in shell egg samples. For RTE products, 4.8% (3/62) were contaminated with *Salmonella* spp., and all isolates were recovered from sausages (8.1%; 3/37). No bacteria were isolated in ham and salami samples. It was found that 29.4% (10/34) of the raw retail chicken samples were contaminated with *Campylobacter* spp. strains. Statistical analysis showed that the percentage of *Salmonella* spp. isolates in raw food samples was significantly higher (*p* = 0.034) than that observed in RTE foods. In addition, a significantly higher *Salmonella* spp. detection rate was recorded in pork compared with chicken meat (*p* = 0.002).

A total of 11 *Salmonella* serotypes were recorded. Their distribution according to the tested food samples is presented in Table 1. In pork, *Salmonella* Typhimurium (*n* = 9 isolates) was the dominant serotype, followed by *Salmonella* Rissen (*n* = 8), *Salmonella* Infantis (*n* = 6), *Salmonella* Bredeney (*n* = 2), *Salmonella* Derby (*n* = 2), *Salmonella* Brandenburg (*n* = 1), *Salmonella* Enteritidis (*n* = 1), *Salmonella* Gloucester (*n* = 1), *Salmonella* Goldcoast (*n* = 1), *Salmonella* Kottbus (*n* = 1), and *Salmonella* Ruzizi (*n* = 1). Of note, all strains isolated from chicken meat were *Salmonella* Infantis. In shell eggs, *Salmonella* Enteritidis (*n* = 2) and *Salmonella* Infantis (*n* = 1) were identified, whereas the sausages were found to be contaminated with *Salmonella* Typhimurium (*n* = 2) and *Salmonella* Derby (*n* = 1). The identified *Campylobacter* species in the raw chicken meat were *Campylobacter coli* (7/10) and *C. jejuni* (3/10).

**Table 1.** Frequency of Resistance to Antimicrobial Agents of the Isolated *Salmonella* Serotypes and *Campylobacter* spp. According to their Isolation Source

Salmonella serotypes and* Campylobacter^a^*spp. and their origin (no. of isolates)	n (%) of resistant strains to													
	AZM	AMP	CHL	CTX	CIP	CST	ERY	GEN	NAL	SMX	STR	TET	TMP	TGC
Raw pork														
*Salmonella* Typhimurium (9)	7 (77.8)	5 (55.6)	2 (22.2)	—	1 (11.1)	1 (11.1)	—	—	—	4 (44.4)	—	4 (44.4)	—	—
*Salmonella* Rissen (8)	7 (87.5)	2 (25)	4 (50)	—	—	3 (37.5)	—	—	—	2 (25)	—	2 (25)	—	—
*Salmonella* Infantis (6)	5 (83.3)	2 (33.3)	3 (50)	—	6 (100)	—	—	—	5 (83.3)	5 (83.3)	—	5 (83.3)	1 (16.6)	2 (33.3)
*Salmonella* Bredeney (2)	2 (100)	—	—	—	—	2 (100)	—	1 (50)	—	—	—	—	—	—
*Salmonella* Derby (2)	1 (50)	1 (50)	1 (50)	—	1 (50)	—	—	—	1 (50)	1 (50)	—	1 (50)	1 (50)	—
*Salmonella* Brandenburg (1)	1 (100)	—	—	—	—	—	—	—	—	—	—	—	—	—
*Salmonella* Enteritidis (1)	1 (100)	1 (100)	—	—	1 (100)	—	—	—	—	—	—	—	—	—
*Salmonella* Gloucester (1)	1 (100)	1 (100)	—	—	—	—	—	—	—	1 (100)	—	1 (100)	—	—
*Salmonella* Goldcoast (1)	1 (100)	—	1 (100)	—	1 (100)	—	—	—	1 (100)	1 (100)	—	1 (100)	—	1 (100)
*Salmonella* Kottbus (1)	1 (100)	1 (100)	—	—	—	—	—	—	—	—	—	—	—	—
*Salmonella* Ruzizi (1)	1 (100)	—	—	—	—	1 (100)	—	—	—	—	—	—	—	—
Raw chicken														
*Salmonella* Infantis (12)	11 (91.7)	2 (16.7)	5 (41.7)	5 (41.6)	11 (91.7)				12 (100)	10 (83.3)		11 (91.7)		7 (58.3)
Eggshell														
*Salmonella* Enteritidis (2)	2 (100)	—	—	—	—	—	—	—	—	—	—	—	—	—
*Salmonella* Infantis (1)	1 (100)	1 (100)	—	—	1 (100)	—	—	—	1 (100)	1 (100)	—	1 (100)	—	1 (100)
Sausage														
*Salmonella* Typhimurium (2)	2 (100)	1 (50)	—	1 (50)	1 (50)	—	—	—	1 (50)	2 (100)	—	2 (100)	1 (50)	2 (100)
*Salmonella* Derby (1)	1 (100)	1 (100)	1 (100)	1 (100)	—	—	—	—	—	1 (100)	—	—	1 (100)	—
Raw chicken														
*Campylobacter coli* (7)	—	—	—	—	5 (71.4)	—	1 (14.3)	—	5 (71.4)	—	1 (14.3)	1 (14.3)	—	—
*C*. *jejuni* (3)	—	—	—	—	3 (100)	—	—	—	3 (100)	—	—	3 (100)	—	—

^a^ Only seven antibiotics were tested.

—, no resistance was recorded; AZM, azithromycin; AMP, ampicillin; CHL, chloramphenicol, CTX, cefotaxime; CIP, ciprofloxacin; CST, colistin; ERY, erythromycin; GEN, gentamicin; NAL, nalidixic acid; SMX, sulfamethoxazole; STR, streptomycin; TET, tetracycline; TMP, trimethoprim; TGC, tigecycline.

The exhibited antimicrobial susceptibility profile of all isolates according to their sample origin is given in Table 1. Of the *Salmonella* strains, 92.2% (47/51) showed resistance to one or more (pork origin *Salmonella* strains to 1–8 agents; chicken—to 3–8 agents; shell eggs—to 2 and 7 agents; sausage—5 and 8 agents, respectively) of the tested 14 antimicrobials, resulting in the expression of a total of 25 resistance profiles. Resistance to AZM (88.2%) was the most common, followed by that to TET (54.9%), SMX (54.9%), CIP (45.1%), NAL (43.1%), AMP (35.3%), CHL (33.3%), TGC (25.5%), CTX (13.7%), CST (13.7%), TMP (7.8%), and GEN (2%). None of the isolates were resistant to MEM and CAF. Regarding the most commonly three encountered serotypes, all the *Salmonella* Infantis (*n* = 19) and the majority (63.6%, 7/11) of *Salmonella* Typhimurium isolates showed MDR, whereas among *S.* Rissen only three (37.5%, 3/8) strains exhibited MDR profile. The other eight less frequently occurring serotypes exhibited a highly variable (to 1–8 agents) resistance pattern.

Antimicrobial susceptibility tests of the *Campylobacter* isolates revealed that all *C. jejuni* strains were MDR to a single triple combination, whereas from the *C. coli* strains only one (14.3%, 1/7) was MDR (Table 1).

## Discussion

This survey generated preliminary results on the distribution of *Salmonella* spp. in raw and RTE foods, and *Campylobacter* spp. in the retail chicken meat collected in two counties of Transylvania, the central historical region of Romania. Our study revealed that *Salmonella* was identified in 22.6%, 9.1%, and 6.3% of the pork, chicken, and shell egg samples, respectively, with an overall prevalence of 14.7% among all screened raw food samples. These results highlight that the investigated animal-derived foods may constitute a potential public health risk. The occurrence of *Salmonella* spp. in fresh raw meat has been previously confirmed in several studies conducted worldwide [reviewed by Baer *et al.* (2013) and Antunes *et al.* (2016)]. Of them, investigations with similar designs to our study highlighted different contamination levels in the Czech Republic [2.7% in pork and 13.6% in chicken (Myšková and Karpišková, 2017)], Germany [0.4% in pork and 17.0% in chicken (Schwaiger *et al.*, 2012)], or the Republic of China [7.1% in pork and 22.5% in chicken (Ren *et al.*, 2017); 63.6% in chicken and 73.1% in pork (Zhang *et al.*, 2018)]. In Romania, previously published research data showed a variable *Salmonella* detection rate for raw chicken [13.2% (Tîrziu *et al.*, 2015); 22.8% (Mihaiu *et al.*, 2014); 4.2% (Dan *et al.*, 2014)] and pork [19.7% (Morar *et al.*, 2015); 23.1% (Mihaiu *et al.*, 2014)] samples.

The recorded significantly higher (*p* = 0.002) *Salmonella* contamination level registered in pork, compared with chicken in this study is in contrast with the results published by several authors (Schwaiger *et al.*, 2012; Ren *et al.*, 2017; Myšková and Karpišková, 2017), but caution should be taken in comparing these results because differences in study design (e.g., sample size, sampling methodology and season, and period), detection methods, or different processing technologies of the raw material may be considered sources of variation of the recorded *Salmonella* prevalence. The isolation of *Salmonella* from shell eggs (6.3%) is a common finding, which has been previously pointed out by many authors (reviewed by Galiș *et al.*, 2013), and serves as a risk factor for cross-contamination of other foods, especially during their household preparation by the consumers.

Of the RTE-examined products, only sausages (8.1%) were found to be *Salmonella* positive. Nonetheless, in the most recent EU summary report, no *Salmonella* data were reported from Romania at the retail level from RTE foods (European Food Safety Authority, 2018). To the authors' knowledge, this is the first published report on the occurrence of this pathogen in RTE foods in Romania. Similar to our findings, the presence of *Salmonella* has been confirmed in sausages in France producing foodborne infections nationwide (Bone *et al.*, 2010). Applied heat treatments, smoking procedures, and the presence of ingredients in RTE products can support the significantly lower (*p* = 0.034) *Salmonella* detection rate in these foods compared with raw foods. Nevertheless, the findings highlight a possible undercooking, contamination from raw materials, or food handlers of RTE products (Baer *et al.*, 2013).

The recorded dominance of *Salmonella* Typhimurium [reported as the most frequently isolated serotype from humans in Romania (European Food Safety Authority, 2019)] and *Salmonella* Rissen in pork and *Salmonella* Enteritidis in shell eggs, and presence of *Salmonella* Infantis exclusively in chicken meat is in agreement with the current knowledge (Baer *et al.*, 2013; Galiș *et al.*, 2013; Antunes *et al.*, 2016, Zahng *et al.*, 2018), according to which all these serotypes are typical of the tested products. Other rare serotypes, such as *Salmonella* Gloucester, *Salmonella* Goldcoast, and *Salmonella* Kottbus were recorded for the first time in Romania, emphasizing the spreading of sporadic serovars in our country.

In our study, of the 51 tested *Salmonella* strains, 92.2% were resistant to at least one antibiotic. Moreover, 68.6% (35/51) exhibited multidrug resistance. Only four pork origin strains were susceptible to all tested drugs (Table 1). No notable associations were recorded between the expressions of the antibiotic resistance patterns of the tested *Salmonella* strains and their isolation source. High resistance levels were recorded to AZM (88.2%), TET (54.9%), SMX (54.9%), CIP (45.1), and NAL (43.1%). These resistance rates (except AZM, for which this is the first published evaluation report in Romania) are consistent with previous reports for both chicken (Tîrziu *et al.*, 2015) and pork (Mihaiu *et al.*, 2014) origin *Salmonella* strains, and reflect their overusage in veterinary medicine.

Similar to our results, increasing AMR trends were published for TET in China [87.5% (Ren *et al.*, 2017); 75.3% (Zhang *et al.*, 2018)], Thailand [73.3% (Sinwat *et al.*, 2015)], North Vietnam [58.5% (Thai *et al.*, 2012)] and the Czech Republic [100% (Myšková and Karpišková, 2017)]; for SMX in Thailand (Sinwat *et al.*, 2015) and Latvia (Terentjeva *et al.*, 2017); for CIP in Latvia [24.0% (Terentjeva *et al.*, 2017)]; and for NAL in North Vietnam [28.8% (Thai *et al.*, 2012)]. Even if the recorded resistance level to another seven antibiotics, including AMP (35.3%), CHL (33.3%), TGC (25.5%), CTX (13.7%), CST (13.7%), TMP (7.8%), and GEN (2%) were moderate or low, the obtained results underscored a wide and worrying resistance spectrum of the *Salmonella* strains. This aspect together with the occurrence of the MDR strains (*n* = 35) in different combination forms (*n* = 21) highlight an urgent need for the implementation of efficient antimicrobial stewardship programs in animal husbandry. On the contrary, the lack of AMR toward MEM and CAF can constitute a promising tool for clinicians in the management of human salmonella infections.

The detection rate of *Campylobacter* spp. in the raw chicken meat in this study was found to be 29.4%, which is <37.4%, the overall frequency of isolation from broiler meat reported by 18 EU member states (European Food Safety Authority, 2018). Compared with several other countries, our prevalence was lower than that reported in India [38.6% (Khan *et al.*, 2018)], Iran [63% (Taremi *et al.*, 2006)], or Republic of Korea [68.3% (Han *et al.*, 2007)], but higher than that obtained in a previous survey in Romania [15.3% (Dan *et al.*, 2015)]. These findings together with the exclusive detection of the two major human pathogens (*C. jejuni* and *C. coli*) in this survey highlight that raw chicken meat can greatly contribute to the human campylobacteriosis cases, the most commonly reported foodborne infections in the EU since 2005.

Among the six tested antimicrobials, a high degree of resistance to CIP (80%), NAL (80%), and TET (40%) were found. Only one strain (10%) was resistant to STR and ERY, and all strains were susceptible to GEN. These resistance levels are higher than those that had been previously recorded for chicken origin *Campylobacter* strains in Romania [31.8% for TET, 9.1% for CIP and NAL, 0% for STR (Dan *et al.*, 2015)]. Different resistance levels were reported for these drugs in other studies conducted in several countries, such as Northern India [59.4%—TET, 6.9%—CIP and NAL—(Khan *et al.*, 2018)], Republic of Korea [99.1—TET, 92.2—CIP and NAL (Han *et al.*, 2007)], or Iran [45.8%—TET, 69.4%—CIP and 75%—NAL (Taremi *et al.*, 2006)]. No data are available on the AMR profile of human origin *Campylobacter* isolates in the most recent EU summary report (European Food Safety Authority, 2019). The occurrence of MDR strains, as in the case of *Salmonella* isolates, could reflect the urgent adaptation and strengthening of guidelines for the prudent use of antimicrobials during poultry production.

## Conclusions

The results of this study showed that the investigated animal-derived foods from Transylvania region, Romania, can harbor MDR *Salmonella* and *Campylobacter* strains, constituting a potential public health risk. Likewise, the findings could reflect an urgent need for the implementation of efficient antimicrobial stewardship programs in animal husbandry, and indirectly can constitute a promising tool for clinicians in the management of human salmonella and campylobacter infections. Further investigations are recommended to a better understanding of the complex puzzle of AMR phenomenon of the foodborne pathogens in our country.
